# P04.22. Comparison of patient-centeredness and patient-reported health outcomes in integrative medicine hospitals with conventional hospitals in Germany

**DOI:** 10.1186/1472-6882-12-S1-P292

**Published:** 2012-06-12

**Authors:** C Scheffer, F Edelhäuser, D Tauschel, A Längler, M Neumann

**Affiliations:** 1 University of Witten / Herdecke, ICURAM, Witten, Germany

## Purpose

Integrative Medicine has been practiced in different hospitals in Germany for several decades. For example, several anthroposophic hospitals integrate conventional medicine with methods including the use of natural remedies, rhythmic embrocations, art therapy and biographic counseling. Moreover, patient-centeredness is a fundamental therapeutic approach especially in Integrative Medicine and many studies have shown significant positive influences on patient-reported physical and psychosocial outcomes. Thus, the purpose of our study is to compare patient-reported health outcomes and patient–provider interaction of integrative medicine hospitals (IMH) with those of conventional hospitals.

## Methods

The Picker Inpatient Questionnaire (PIQ) was used to get feedback from patients on the quality of care received in the IMH and conventional hospital over a period of 10 years (2000-2009). In the PIQ patients were asked to report their experience with specific events and processes in the hospital. The survey includes questions on the patient-provider interaction (e.g. patient-physician-interaction, patient-nursing-interaction, family involvement) and patient reported health outcomes (e.g. medical treatment success, complications after discharge, improvement of main complaints). Problem frequency of items and subscales are compared with a patient control group of matched pairs treated in conventional hospitals. Matched pairs were created according to several criteria of the hospital (same discipline, similar size of department) and of the patient (age, gender, education, patients´ self-assessment about their health status and presence of pain). The Mann-Whitney-Test was used for comparison with control groups.

## Results

The PIQ was presented to N=5391 patients of several IMH reporting about patient-provider interaction and health outcomes compared with matched pairs of conventional hospitals. Preliminary evaluation showed a lower problem frequency patient-physician-interaction and patient-nursing-interaction in IMH.

## Conclusion

According to patients’ subjective feedback captured by the PIQ, patient-centeredness of IMH seems to be higher than in conventional hospitals.

